# Epoxypukalide Induces Proliferation and Protects against Cytokine-Mediated Apoptosis in Primary Cultures of Pancreatic β-Cells

**DOI:** 10.1371/journal.pone.0052862

**Published:** 2013-01-02

**Authors:** José Francisco López-Acosta, José Luis Moreno-Amador, Margarita Jiménez-Palomares, Ana R. Díaz-Marrero, Mercedes Cueto, Germán Perdomo, Irene Cózar-Castellano

**Affiliations:** 1 Instituto de Genética y Biología Molecular (IBGM)-Universidad de Valladolid, Valladolid, Spain; 2 Unidad de Investigación, Hospital Universitario Puerta del Mar, Cádiz, Spain; 3 Instituto de Productos Naturales y Agrobiología del CSIC, La Laguna, Tenerife, Spain; CRCHUM-Montreal Diabetes Research Center, Canada

## Abstract

There is an urgency to find new treatments for the devastating epidemic of diabetes. Pancreatic β-cells viability and function are impaired in the two most common forms of diabetes, type 1 and type 2. Regeneration of pancreatic β-cells has been proposed as a potential therapy for diabetes. In a preliminary study, we screened a collection of marine products for β-cell proliferation. One unique compound (epoxypukalide) showed capability to induce β-cell replication in the cell line INS1 832/13 and in primary rat cell cultures. Epoxypukalide was used to study β-cell proliferation by [^3^H]thymidine incorporation and BrdU incorporation followed by BrdU/insulin staining in primary cultures of rat islets. AKT and ERK1/2 signalling pathways were analyzed. Cell cycle activators, cyclin D2 and cyclin E, were detected by western-blot. Apoptosis was studied by TUNEL and cleaved caspase 3. β-cell function was measured by glucose-stimulated insulin secretion. Epoxypukalide induced 2.5-fold increase in β-cell proliferation; this effect was mediated by activation of ERK1/2 signalling pathway and upregulation of the cell cycle activators, cyclin D2 and cyclin E. Interestingly, epoxypukalide showed protection from basal (40% lower versus control) and cytokine-induced apoptosis (80% lower versus control). Finally, epoxypukalide did not impair β-cell function when measured by glucose-stimulated insulin secretion. In conclusion, epoxypukalide induces β-cell proliferation and protects against basal and cytokine-mediated β-cell death in primary cultures of rat islets. These findings may be translated into new treatments for diabetes.

## Introduction

Diabetes is one of the most devastating diseases of our era. The most common forms are type 1 and type 2 diabetes. β-cell mass decreases approximately by 70–100% in type 1 diabetes and up to 65% in type 2 diabetes. Both forms of the disease display increased β-cell apoptosis [1], [2].

Finding therapeutic targets and molecules that preserves and/or enhances functional beta-cell mass is essential in the long-term treatment of diabetes. It is known that adult pancreatic β-cells, in rodents and humans, are generated from the proliferation of differentiated beta-cells [3], [4]. Some growth factors have been shown to induce β-cell proliferation in vitro and in vivo, among them HGF (hepatocyte growth factor), PL (placental lactogen) and PTHrP (Parathyroid hormone related protein) have also shown enhancement of β-cell survival [5].

Classical signalling pathways involved in beta-cell proliferation comprise PI3K/AKT, ERK1/2, PKC and JAK2/STA5 [5]. Activation of these intracellular effectors ends on the regulation of the proteins that control G1/S interphase of the cell cycle. Among all the proteins that regulate this interphase, pRb is the key checkpoint gatekeeper. The complexes that regulate pRb phosphorylation (and its inactivation to allow cell cycle progression) are cdk4/cyclin D and cdk2/cyclinE [6]. Both, cyclin D2 and cyclin E have been found to have a relevant role in several models of β-cell proliferation [5], [7], [8], [9], [10], [11], [12], [13], being essential to induce β-cell replication.

Epoxypukalide was reported by Schmitźs group in 1984 by the first time [14], they purified it from the gorgonian *Leptogorgia setacea*. Epoxypukalide and other furanocembranolides were identified in 2007 by Dorta et al. [15] who searched for marine natural products in benthic species around the Isthmus of Panama. Finally Grote et al. [16] described a group of furanocembranoids isolated from the soft coral *Sinularia asterolobata*, being one of them epoxypukalide. They studied the cytotoxic and anti-proliferative effect of those compounds, reporting that epoxypukalide did not inhibit HeLa (human epithelial carcinoma) cell proliferation nor produces cytotoxicity in the cell lines L-929 (murine fibrosarcoma) or K-562 (human erythroleukemic cell line) [16].

In the current report, we demonstrate a proliferative effect of epoxypukalide on primary cultures of rat β-cells, in parallel to a protective effect on β-cell death while preserving β-cell function. Epoxypukalide-induced β-cell proliferation is mediated by ERK1/2 activation and targets cyclin D2 and cyclin E. Taken together these data support a potential use of epoxypukalide in the treatment of diabetes.

## Methods

### Ethical Approval

Experimental procedures were approved by the Animal Care and Use Committee of the University of Cadiz in accordance with the Guidelines for Care and Use of Mammals in Research (European Commission Directive 86/609/CEE and Spanish Royal Decree 1201/2005).

### Cell Culture

INS-1 832/13 cells were grown at 37°C and 5% CO2 in a humidified atmosphere. INS-1 culture medium was RPMI-1640 with 2 mmol/l L-glutamine supplemented with 11 mmol/l D-glucose, 10% fetal bovine serum, 100 U/ml penicillin, 100 µg/ml streptomycin, 10 mmol/l HEPES, 1 mmol/l sodium pyruvate, and 50 µmol/l β-mercaptoethanol.

Rat islets were isolated from 2 months old male Wistar rats provided by Animal Production and Experimentation Service (SEPA, University of Cádiz) by a standard procedure and incubated at 37°C and 5% CO2 in a humidified atmosphere. Culture medium was RPMI-1640 with 2 mmol/l L-glutamine supplemented with 5.5 mmol/l D-glucose, 10% fetal bovine serum, 100 U/ml penicillin, and 100 µg/ml streptomycin.

### Proliferation in INS-1 Cells

For the first screening, INS-1 832/13 cells were seeded at a density of 20,000 cells per well in 96-well plates. Natural products were assayed at a final concentration of 0.1 µM in culture medium with 5.5 mM glucose. Proliferation was measured after 24 hours by BrdU kit (Roche, Germany), following manufacturer’s instructions.

### Proliferation in Rat Islets

Rat islets were isolated and incubated in serum free medium with 0, 0.01, 0.1 and 1 µM epoxypukalide after an overnight recovery. 24 hours later, 100 IEq (islet equivalent) per well were seeded in 24-well plates with fresh medium without FBS containing [^3^H]thymidine (1 µCi/well). [^3^H]thymidine incorporation was measured 24 h later; radioactivity was corrected for protein levels measured by the BCA kit (Thermo Fisher, USA). Results are expressed as percentage of counts per minute incorporated per microgram of protein in control cells (100%).

To determine specific β-cell proliferation, isolated rat islets were treated with medium without serum containing 0.1 µM epoxypukalide after an overnight recovery. 24 hours later, islets were serum depleted and incubated with 1 µl/ml BrdU (Sigma-Aldrich, USA). Islets were then incubated for 24 hours. Afterwards islets were fixed with Bouińs Solution (Sigma-Aldrich, USA) for 1 hour and then with formalin until treated for paraffin blocks. 5 µm sections were stained with rat anti-BrdU antibody (Abcam, UK) and with guinea pig anti-Insulin antibody (Invitrogen, USA). Fluorescence images of the sections were acquired using a Olympus BX40 fluorescence axial microscope. The BrdU-positive nuclei of beta cells and the total nuclei of beta cells were counted manually with the assistance of the ImageJ software. At least 1000 insulin-positive cells for each preparation were counted.

### Western-blot

Islets used to study signalling pathways activation (AKT and ERK1/2) were treated for 0, 5, 15 or 30 minutes with 0.1 µM epoxypulide after overnight recovery from islet isolation.

Islets used to study cell cycle proteins expression were treated for 24 h with 0.1 µM epoxypukalide after overnight recovery from islet isolation.

After their treatment, islets were washed with PBS (phosphate-buffered saline) and lysed in lysis buffer (125 mM Tris, pH 6.8, 2% SDS, 1 mM DTT and protease/phosphatase inhibitors). The protein lysates were briefly sonicated and centrifuged for 1 minute at the maximum speed. Proteins were measured by Micro BCA kit (Thermo-Fisher, USA), run on a 10% EZ-Run Gel (Fisher Scientific, USA) and then transferred to a PVDF Immobilon-P membrane (Millipore, USA). Blots were incubated with the following antibodies: anti-Phospho-AKT (Ser473) (Cell Signaling, UK), anti-phospho-p44/42 MAPK (ERK1/2) (Thr202/Tyr204) (Cell Signaling, UK), anti-cyclin D1 (Invitrogen, USA), anti-cyclin D2 (Invitrogen, USA), anti-cyclin D3 (Abcam, UK), anti-cyclin E (Santa Cruz, USA), anti-cdk4 (Abcam, UK), anti-cdk2 (Santa Cruz, USA) or anti-actin (Sigma, USA).

For cleaved caspase 3 western-blots, rat islets were treated with cytokinés mix (IL-1β, IFN-γ and TNF-α) 1000 U/mL. Islets were preincubated with epoxypukalide 0.01, 0.1 and 1 µM for 48 h. Western-blot was performed as described above using anti-cleaved-caspase 3 antibody (Cell Signaling, UK).

### Beta-cell Death

5 µm sections obtained as explained above were used for TUNEL-staining. DeadEnd Fluorometric Tunel System Kit (Promega, USA) was used following manufacturer’s instructions. TUNEL-positive/beta-cells were counted and represented as a percentage of total number of beta-cells. Nuclei of beta cells were counted manually with the assistance of the ImageJ software. At least 1000 TUNEL-positive cells for each preparation were counted.

### Glucose-stimulated Insulin Secretion and Insulin Content

Secretion assays was performed on treated rat islets and their controls. Islets were plated on cell culture inserts onto 24-well plates at a density of 20 IEq groups in HEPES balanced salt solution (HBSS) (114 mmol/l NaCl, 4.7 mmol/l KCl, 1.2 mmol/l KH2PO4, 1.16 mmol/l MgSO4, 20 mmol/l HEPES, 2.5 mmol/l CaCl2, 25.5 mmol/l NaHCO3, and 0.2% bovine serum albumin [essentially fatty acid free], pH 7.2). Islets were washed twice in 1 ml HBSS with 2.2 mmol/l glucose followed by 10 minutes of preincubation in 2 ml of the same buffer. Insulin secretion was stimulated by using static incubation for 30 minutes in 1 ml of the same buffer, followed by 30 minutes of incubation in HBSS containing 22 mmol/l glucose. Secretion samples were measured by ultra-sensitive rat insulin ELISA (Alpco Diagnostics, USA).

20 IEq by triplicate of each condition (control, 0.01, 0.1 and 1 µM epoxypukalide) were harvested after 24 h treatment. Insulin was extracted using 1 mL of acid-ethanol (70% ethanol and 0.02% HCl). Insulin was measured by rat insulin ELISA.

### Statistics

Statistical analyses of data were performed by Student-t test when two conditions were compared and ANOVA when more than two conditions were compared. Data were expressed as mean ± SD. P values <0.05 were considered significant.

## Results

### Screening of a Collection of Marine Natural Products

**Figure 1:**
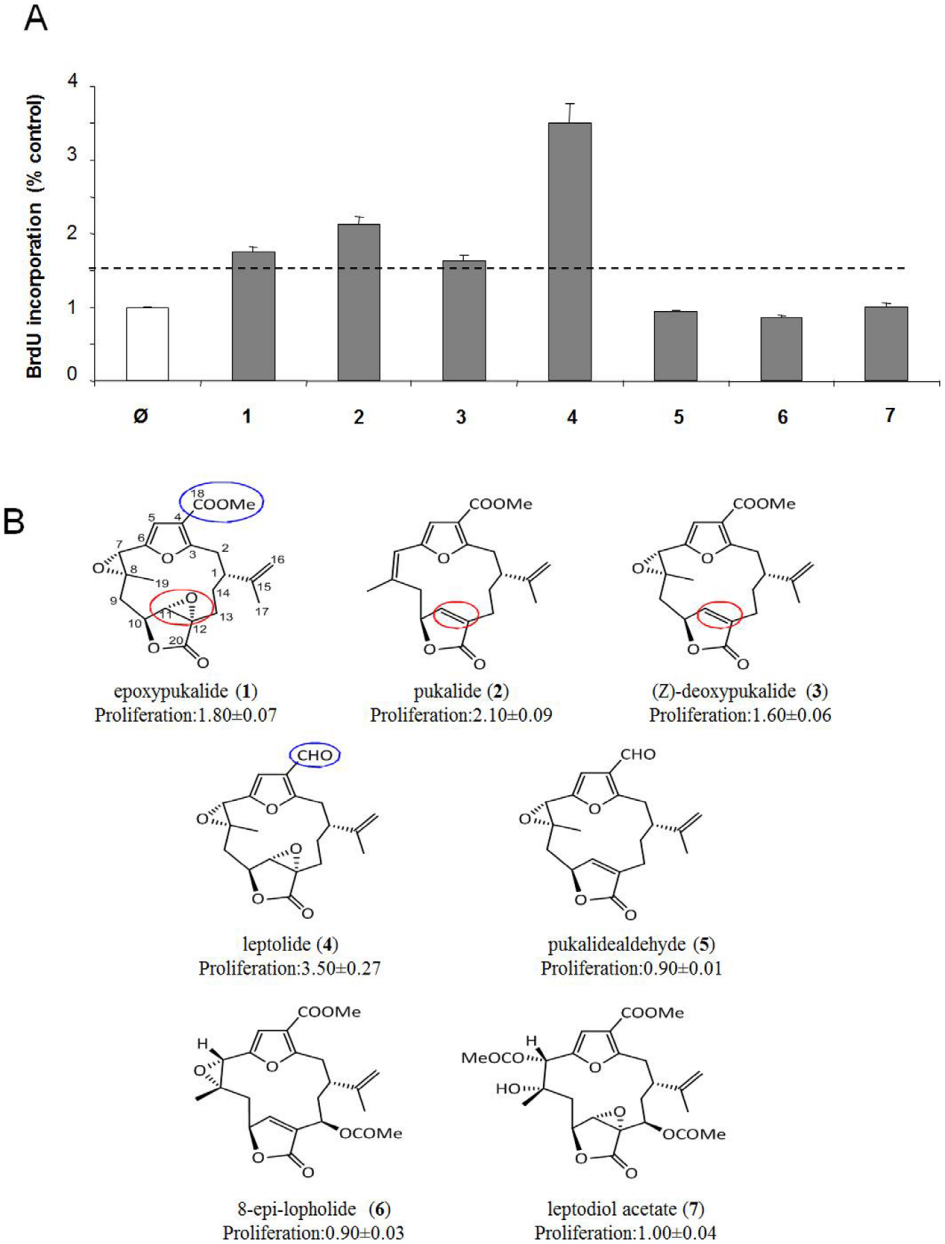
Screening of a collection of marine natural products. INS-1 832/13 cells were preincubated with 0.1 µM of the seven furanocembranolides (1–7) and proliferation was measured by BrdU incorporation as described in Methods section (N = 6). Proliferation was defined as the fold change above untreated cells (1.0) Threshold was over 1.5-fold increase in proliferation (A). Chemical structure of the seven furacembranolides (B).

Seven furanocembranolides (Figure 1) obtained from the soft coral *Leptogorgia* spp. [15] were screened for induction of β-cell proliferation in the rat cell line INS-1 832/13. This group of compounds was chosen from a larger collection of marine natural products, after a preliminary screening, being the unique group of compounds inducing beta-cell proliferation of the INS1 832/13 cells (data not shown). Cell proliferation was measured using BrdU incorporation. Four products, epoxypukalide (**1**), pukalide (**2**), (*Z*)-deoxypukalide (**3**) and leptolide (**4**), induced β-cell proliferation over the threshold (≥1.5-fold compared to control) (Figure 1).

### Epoxypukalyde Induces β-cell Proliferation in Primary Rat Islets

**Figure 2:**
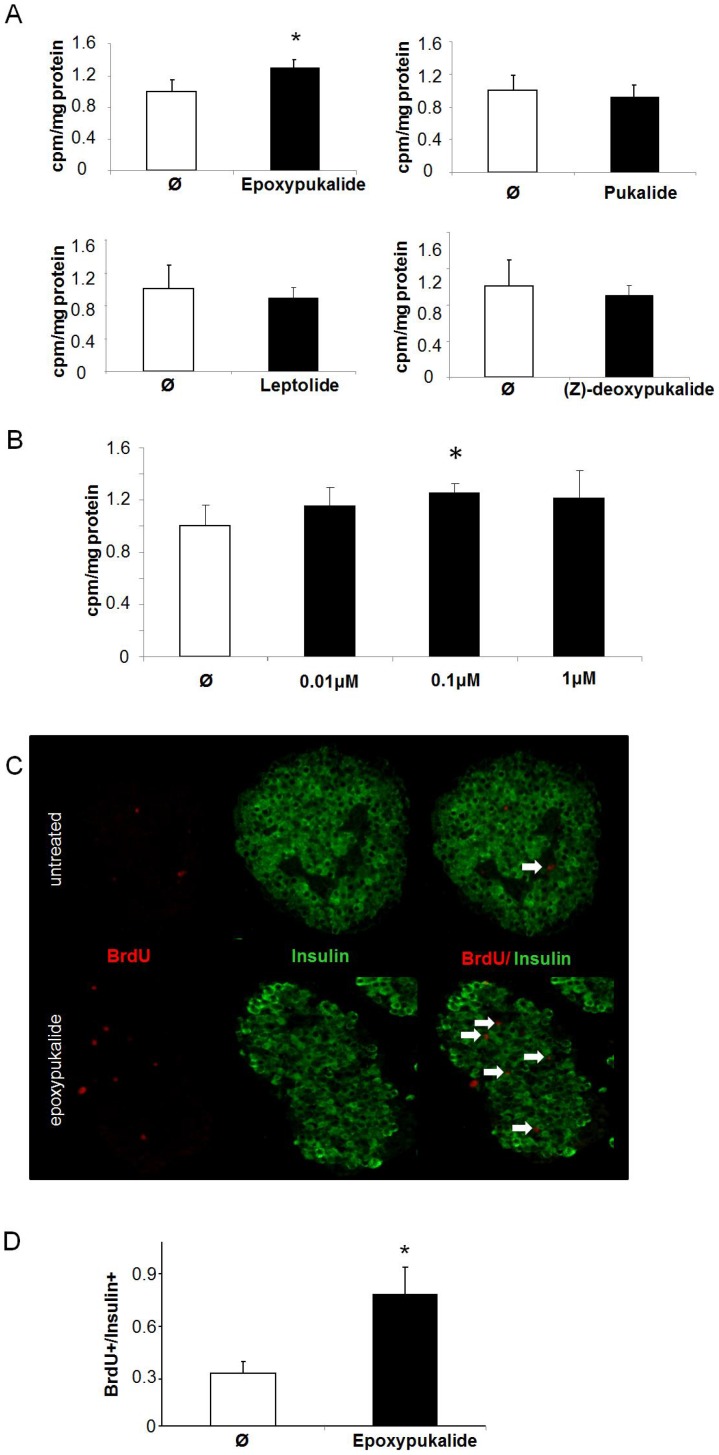
Epoxypukalyde induces β-cell proliferation in primary rat islets. Primary cultures of rat islets were treated with 0.1 µM epoxypukalide, pukalide, leptolide, (Z)-deoxypukalide or vehicle (Ø) for 24 h. Cell proliferation was measured by [^3^H]thymidine incorporation as described in Methods section (N = 6 in triplicate) (*p<0.05) (A). Dose-dependence experiments to test epoxypukalide effectiveness in cell proliferation in rat islets (N = 6–12 in triplicate). Proliferation was measured by [^3^H]thymidine incorporation (B). Representative pictures of primary islet cell cultures treated with 0.1 µM epoxypukalide for 24 h, sections were stained for insulin (green) and BrdU (red). Arrows indicate BrdU-positive β-cells (C). Quantification of the percentage of BrdU-positive β-cells (N = 6) (*p<0.05) (D).

We used isolated rat islets to test ex-vivo the proliferative capability of compounds **1–4**. Proliferation was measured using [^3^H]thymidine incorporation. Epoxypukalide (**1**) showed an increase of 30% in proliferation compared to control, conversely pukalide (**2**), leptolide (**4**) and (*Z*)-deoxypukalide (**3**) did not induce proliferation in primary cell cultures (Figure 2A). Then, we performed a dose response evaluation of epoxypukalide to test cell proliferation, using concentrations from 0.01–1 µM. Only 0.1 µM showed a significant increase in cell proliferation (Figure 2B). Thus, for following experiments involving beta-cell proliferation we have used 0.1 µM epoxypukalide. As a second approach and in order to measure specific β-cell proliferation, we used BrdU incorporation followed by insulin/BrdU staining to quantify BrdU+insulin+ cells in respect to total insulin+ cells. Epoxypukalide treatment showed 250% increased β-cell proliferation compared to control cells (Figure 2C, D).

### Activation of Signalling Pathways by Epoxypukalyde in Primary Rat Islets

**Figure 3:**
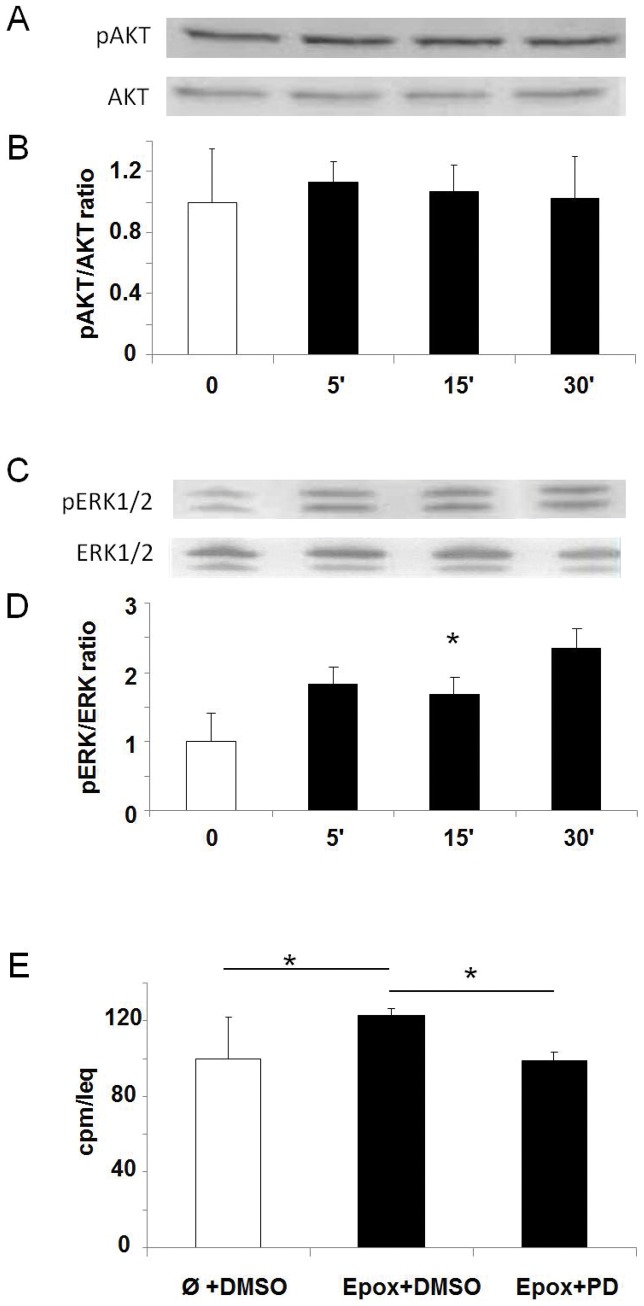
Activation of signalling pathways by epoxypukalyde in primary rat islets. Representative western-blot illustrating the effect of epoxypukalide on AKT proliferation pathway (A). Quantification of pAKT/AKT western-blots (N = 4) (B). Representative western-blot illustrating activation of ERK1/2 pathway (C). Quantification of pERK/ERK western-blots (N = 4) (D). Cell proliferation was induced with epoxypukalide (Epox) in rat islets pretreated in the presence of PD98059 (PD) (inhibitor or ERK1/2 pathway) or vehicle (DMSO). β-cell proliferation was measured by [^3^H]thymidine incorporation assay and compared to control (vehicle-treated islets) (N = 3 in triplicate) (E). (*p<0.05).

The analysis by western-blot of two of the most relevant signalling pathways involved in β-cell proliferation indicated that epoxypukalide did not activate AKT pathway (Figure 3A, B), but ERK1/2 was activated within the first 30 minutes after treatment (Figure 3C, D). To determine whether ERK1/2 could mediate the proliferative effect of epoxypukalide, a specific pharmacological inhibitor of ERK1/2 (PD98059) was used and its effect on epxypukalide-β-cell proliferation was examined using [^3^H]thymidine incorporation. PD98059 decreased the proliferation induced by epoxypukalide to basal levels (Figure 3E), showing that this pathway is involved in epoxypukalide-induced β-cell proliferation.

### Epoxypukalide Induces Expression of Cell Cycle Activators

**Figure 4:**
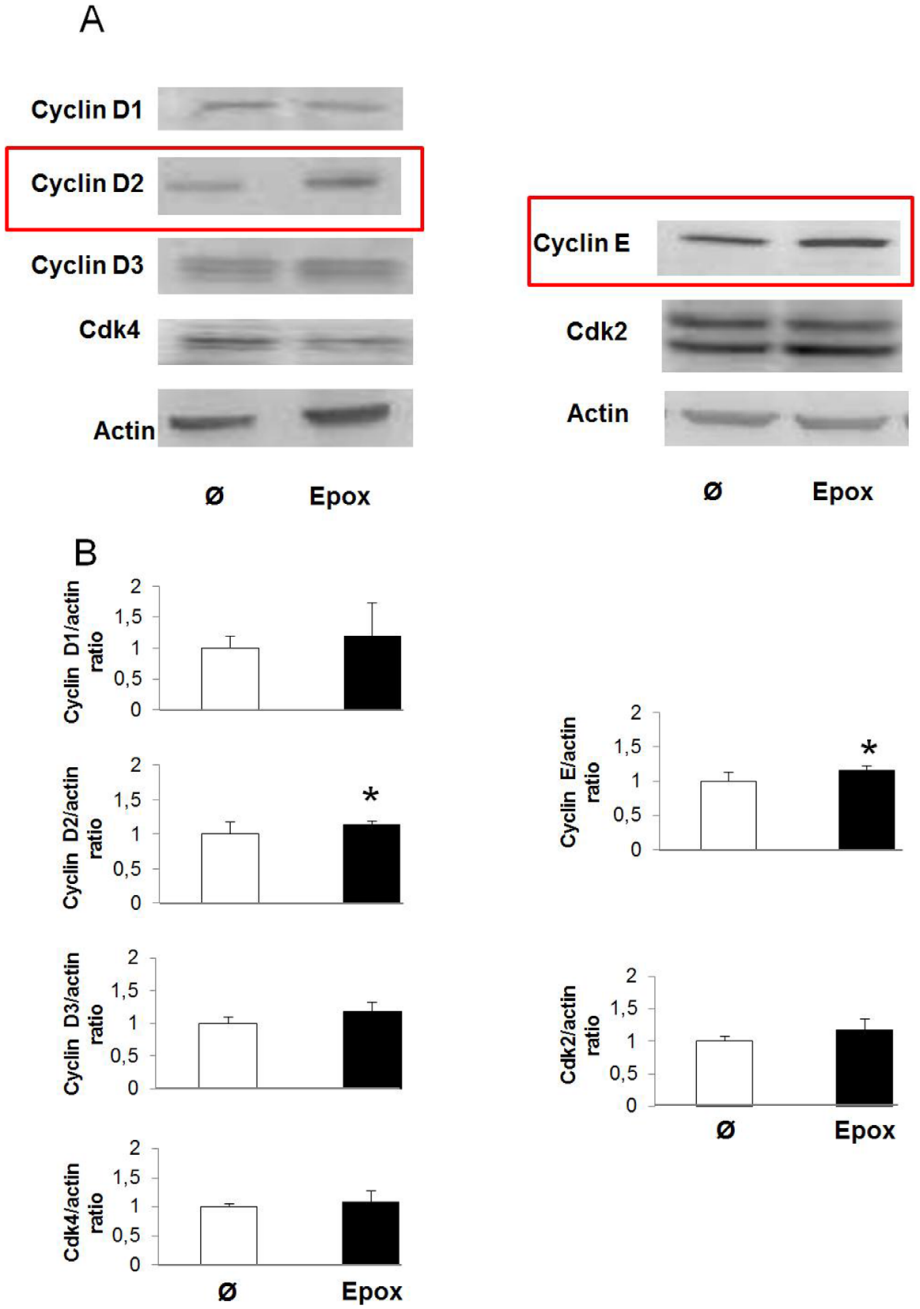
Epoxypukalide induces expression of cell cycle activators. Primary cultures of rat islets were treated with epoxypukalide (Epox) or vehicle. Representative western-blots of cyclin D1, cyclin D2, cyclin D3, cdk4, cyclin E and cdk2 (A). Densitometric analysis of western-blots (N = 3–10) (B). (*p<0.05).

To further investigate the mechanisms by which epoxypkalide induces β-cell proliferation, we performed western-blots for the proteins involved in pRb phosphorylation in G1/S (cyclin D1, cyclin D2, cyclin D3, cdk4, cyclin E and cdk2) in rat islets in the presence or absence of epoxypukalide. Of the six tested proteins, only cyclin D2 and cyclin E were up-regulated, showing ∼15% more expression in epoxypukalide-treated islets compared to control islets (Figure 4A, B).

### Epoxypukalide Protects Basal and Cytokine-induced β-cell Death

**Figure 5:**
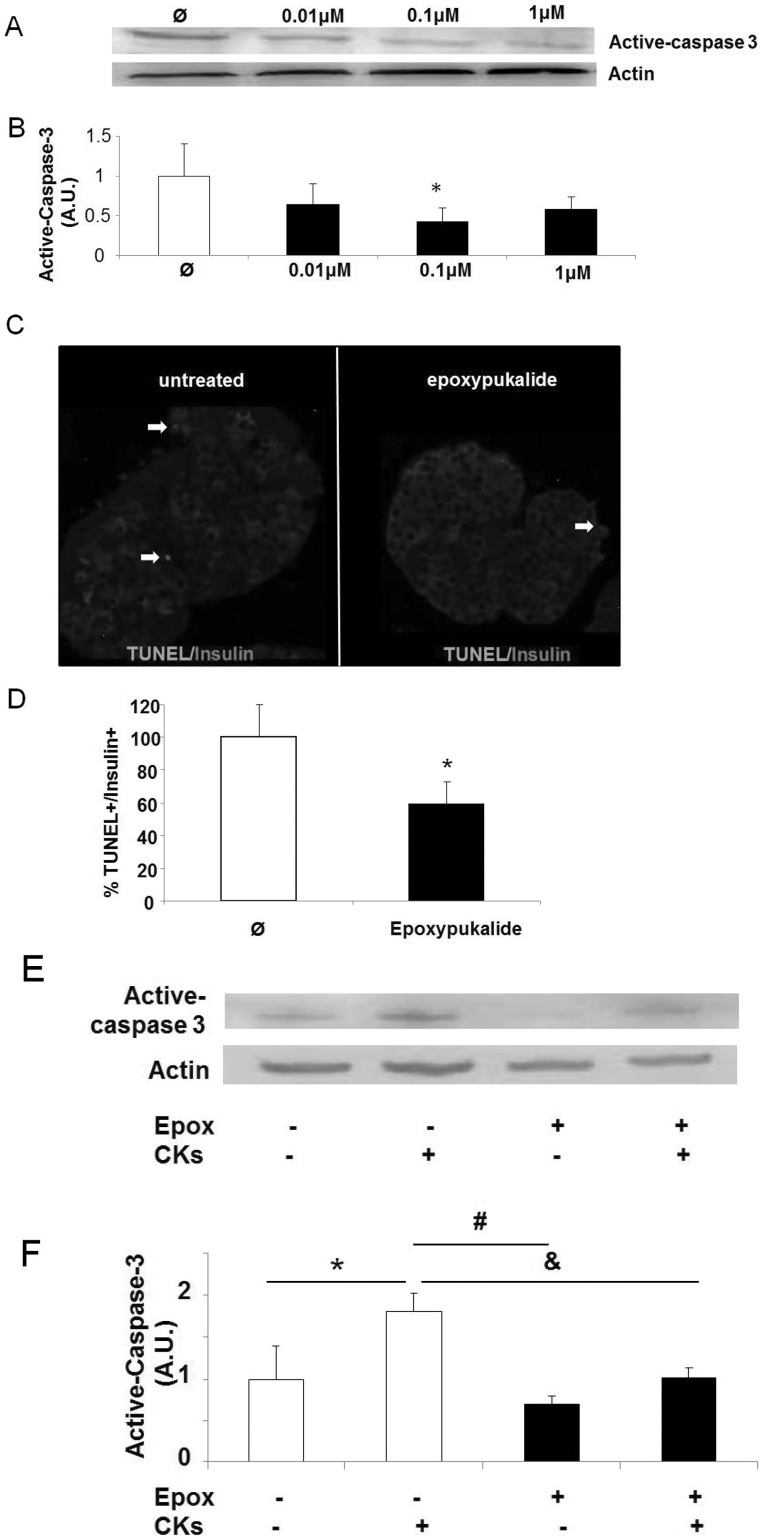
Epoxypukalide protects basal and cytokine-induced β-cell death. Representative western-blot illustrating cell death protection mediated by epoxypukalide in a dose-dependent manner (A). Densitometric analysis of western-blots (N = 4) (B). Representative pictures of primary cultures of rat islets treated with epoxypukalyde (Epox) for 24 h, sections were stained for insulin (red) and TUNEL (green) and arrows indicate TUNEL-positive β-cells (C). Quantification of the percentage of TUNEL-positive β-cells (N = 6) (D). Representative western-blot illustrating the prosurvival effect of epoxypukalide (E). Densitometric analysis of western-blots (N = 5) (F). (#,&,*p<0.05).

To investigate the effects of epoxypukalide on β-cell survival, we performed a dose response evaluation of epoxypukalide, using concentrations from 0.01–1 µM. We tested cell death using caspase 3 activation detected by western-blot. Only 0.1 µM showed a significant decrease in cell death (Figure 5A, B), thus, for following experiments involving beta-cell death we have used 0.1 µM. To confirm epoxypukalide effect on beta-cell death we performed TUNEL/insulin staining on sections of rat islets that were previously exposed to epoxypukalide and their untreated control. After quantification of TUNEL+insulin+ cells, we detected 40% lower basal β-cell death in epoxypukalide treated islets than in control islets (Figure 5C, D). To test epoxypukalide-protective effect in conditions of stress for the pancreatic islets, we treated rat islets with cytokines (CK), in the presence or absence of epoxypukalide, and we determined activation of the apoptotic pathway using caspase-3 activation. Islets treated with CK showed 80% increase in cleaved-caspase 3 compared to control, this effect was reverted when islets were treated with 0.1 µM epoxypukalide (Figure 5E, F).

### Epoxypukalyde does not Impair β-cell Function

**Figure 6:**
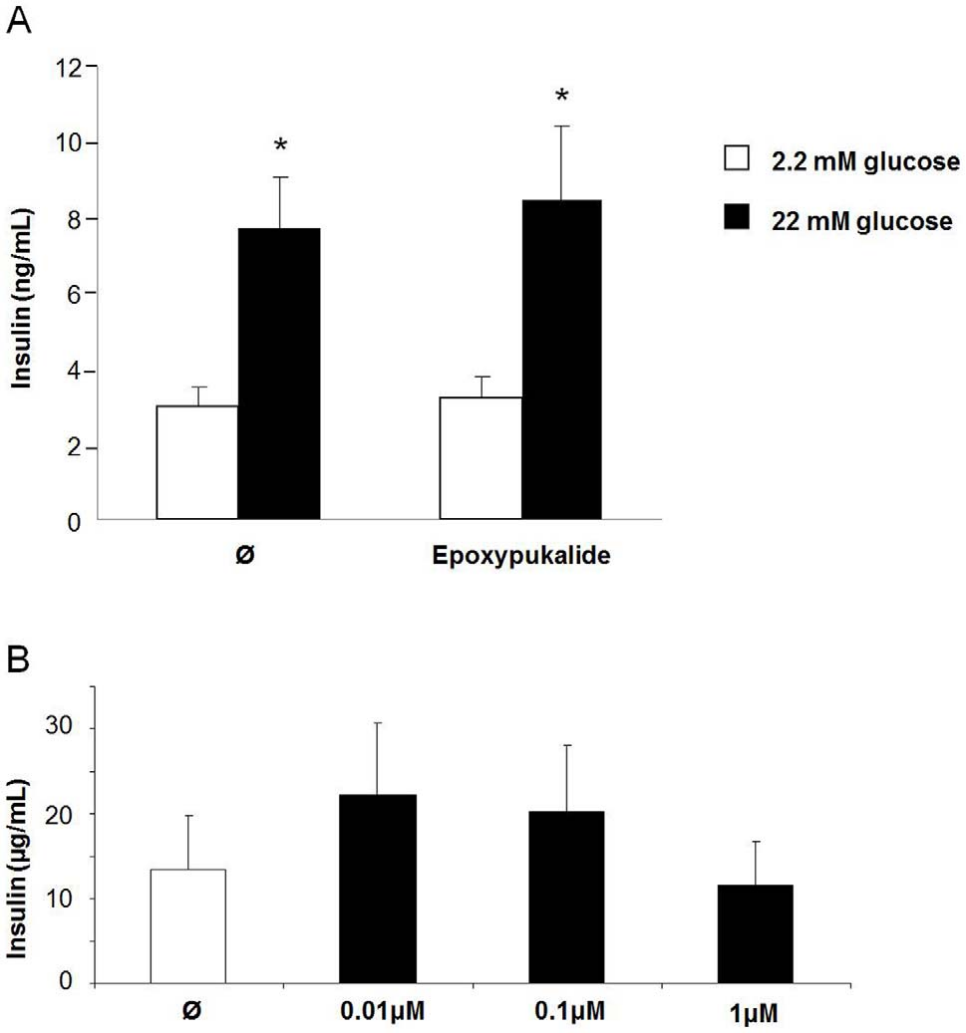
Epoxypukalyde does not impair β-cell function. Glucose-stimulated insulin secretion was performed in 0.1 µM epoxypukalide- or vehicle-treated rat islets as described in Methods section. 5.5 mM glucose (white bar) and 22 mmol/L glucose (black bar). Experiments were performed in triplicate (N = 9) (A). Insulin content was measured in islets treated with different concentrations of epoxypukalide (0.01–1 µM) (N = 5 in triplicate) (B) (*p<0.05).

Finally, we tested whether epoxypukalide alters β-cell function. To this end we performed glucose-stimulated insulin secretion on epoxypukalide-treated rat islets to check for β-cell function in vitro. Insulin secretion after glucose overload was completely normal (Figure 6A). Furthermore we have measured insulin content in control islets and epoxypukalide- treated islets, finding that there are not significant differences between epoxypukalide-treated and control islets (Figure 6B). Both results support that there is not deleterious effect of epoxypukalide on β-cell function.

## Discussion

Search for new molecules to preserve functional β-cell mass is important for diabetes. Research groups and pharmaceutical companies are involved in the chase of new molecules that can induce β-cell proliferation and/or enhance β-cell survival; their searches are based on the detection of activation of signalling pathways involved in β-cell death or β-cell proliferation [17], [18]. The interaction between chemists that purify and identify new compounds and biologists who test their function is essential for the discovery of new treatments.

Accordingly to the structures of compounds 1–4 and the proliferation results, it can be observed that although they possess the same carbon skeleton, differences in their functionalizations are critical to induce proliferation in primary cell cultures. From comparison of epoxypukalide (1) and leptolide (4) it can be deduced that the presence of a methyl ester instead of the alhdeyde group at C-18 is a key to the potency of furanocembranolides. Also from the observation of epoxypukalide (1), pukalide (2) and (Z)-deoxypukalide (3) it can be deduced that epoxydation, especially at C-11−C-12, is crucial to show proliferative activity in primary cell cultures.

From the dose-dependence studies we have learned that 0.1 µM is the optimal concentration to induce beta-cell proliferation and to inhibit beta-cell death. This should be the reference concentration to design in vivo experiments in rodents.

Two of the most relevant pathways involved in β-cell proliferation (AKT and ERK1/2) were studied. Analysis of these two pathways indicated that epoxypukalide did activate ERK1/2 pathway. Some growth factors have shown to induce β-cell proliferation through ERK1/2 signalling including HGF [19], insulin and IGF1 (insulin growth factor-1) [5].

It has been shown that G1/S interphase is essential in the regulation of β-cell cycle proliferation. Cyclin D2 and E, individually, have been shown previously to induce β-cell replication under the stimulus of several mitogens including high glucose, GH (growth hormone), PRL (prolactin) and PTHrP [5], [7], [8], [9], [10], [11], [12], [13]. Cyclin D2 joins to cdk4 and cyclin E to cdk2 maintaining pRb phosphorylation in different residues. We can argue that since both cyclins are upregulated by epoxypukalide, it induces a synergistic effect responsible to maintain beta-cell proliferation, even though their expression levels increase modestly.

CK (cytokine)-induced β-cell apoptosis is a hallmark of diabetes [1], [20], [21], [22]. Our experiments showed epoxypukalide protects β-cells from basal and CK-induced apoptosis. Other small molecules have shown similar effects on protection of CK-induced β-cell apoptosis [18], but their proliferative capability was not tested. It is important to expand β-cell mass in order to have an efficient control of glucose homeostasis in diabetes. In support of this notion, Zhao et al. showed that diabetic mice (leptin receptor null-db/db) treated with a sphingosine 1-phosphate receptor modulator (FTY720) counteracted hyperglycemia by induction of β-cell proliferation and β-cell mass expansion [23].

Increased β-cell proliferation may involve loss of differentiation and cell function failure. Thus, we performed glucose-stimulated insulin secretion on epoxypukalide-treated rat islets to check for β-cell function in vitro. Insulin production and secretion after glucose overload was completely normal, meaning that there is not deleterious effect of epoxypukalide on β-cell function. This is important in order of a possible use of this compound in the treatment of diabetes. In this direction, Grote et al. showed that epoxypukalide was not cytotoxic for L-929 (murine fibrosarcoma) or K-562 (human erythroleukemic cell line) [16]. Their results sustain that epoxypukalide has not adverse effects on other cell types.

In conclusion, our study supports that epoxypukalide is a potential drug for the treatment of diabetes, further work in granted in order to study its in vivo effects.
