# Correction: Epoxypukalide Induces Proliferation and Protects against Cytokine-Mediated Apoptosis in Primary Cultures of Pancreatic β-Cells

**DOI:** 10.1371/annotation/7a2db0c8-6ed3-4d0b-9bff-9c0a8b818b8a

**Published:** 2013-05-28

**Authors:** José Francisco López-Acosta, José Luis Moreno-Amador, Margarita Jiménez-Palomares, Ana R. Díaz-Marrero, Mercedes Cueto, Germán Perdomo, Irene Cózar-Castellano

There was an error with the presentation of Figure 5. Part C was displayed in black and white. The correct image displays the red and green insulin staining, which is viewable at the following link: 

**Figure:**
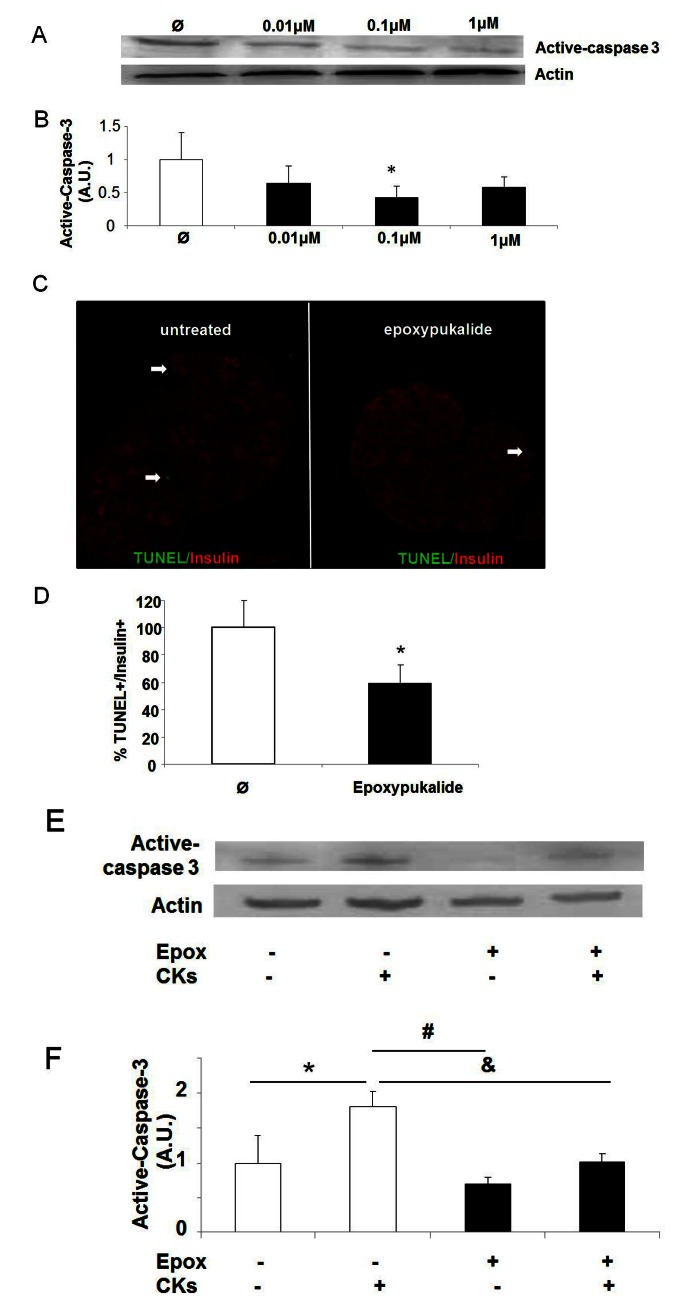


.

